# Unveiling Excitonic Dynamics in High‐Efficiency Nonfullerene Organic Solar Cells to Direct Morphological Optimization for Suppressing Charge Recombination

**DOI:** 10.1002/advs.201802103

**Published:** 2019-02-19

**Authors:** Xiaoyu Liu, Yajie Yan, Alireza Honarfar, Yao Yao, Kaibo Zheng, Ziqi Liang

**Affiliations:** ^1^ Department of Materials Science Fudan University Shanghai 200433 China; ^2^ Department of Chemical Physics and NanoLund Lund University P.O. Box 124 22100 Lund Sweden; ^3^ Department of Physics and State Key Laboratory of Luminescent Materials and Devices South China University of Technology Guangzhou 510640 China; ^4^ Department of Chemistry Technical University of Denmark DK‐2800 Kongens Lyngby Denmark

**Keywords:** charge recombination, film morphology, hole and electron transfer, nonfullerene acceptors, organic solar cells

## Abstract

Nonfullerene acceptors (NFAs)‐based organic solar cells (OSCs) have recently drawn considerable research interests; however, their excitonic dynamics seems quite different than that of fullerene acceptors‐based devices and remains to be largely explored. A random terpolymer of **PBBF11** to pair with a paradigm NFA of 3,9‐bis(2‐methylene‐(3‐(1,1‐dicyanomethylene)‐indanone)‐5,5,11,11‐tetrakis(4‐hexylphenyl)‐dithieno[2,3‐d:2′,3′‐d′]‐s‐indaceno[1,2‐b:5,6‐b′]dithiophene (ITIC) such that both complementary optical absorption and very small offsets of both highest occupied molecular orbital and lowest unoccupied molecular orbital energy levels are acquired is designed and synthesized. Despite the small energy offsets, efficient electron/hole transfer between **PBBF11** and ITIC is both clearly observed from steady‐state photoluminescence and transient absorption spectra and also supported by the measured low exciton binding energy in ITIC. Consequently, the **PBBF11**:ITIC‐based OSCs afford an encouraging power conversion efficiency (PCE) of 10.02%. Although the good miscibility of **PBBF11** and ITIC induces a homogenous blend film morphology, it causes severe charge recombination. The fullerene acceptor of PC_71_BM with varying loading ratios is therefore added to modulate film morphology to effectively reduce the charge recombination. As a result, the optimal OSCs based on **PBBF11**:ITIC:PC_71_BM yield a better PCE of 11.4% without any additive or annealing treatment.

## 1 Introduction

In recent several years, nonfullerene acceptors (NFAs)‐based solution‐processed bulk heterojunction (BHJ) organic solar cells (OSCs) have drawn vast attention with increasingly rapid developments.^1^, ^2^, ^3^, ^4^, ^5^, ^6^, ^7^, ^8^, ^9^, ^10^ In principle, NFAs possess tunable molecular structures, allowing for a fine control of optical bandgap (*E*_g_), energy levels, light absorption, and crystallinity to match well with a variety of organic donor semiconductors. The record power conversion efficiencies (PCEs) in NFAs‐based single‐junction binary OSCs have now reached ≈14%, which has surpassed those of fullerene acceptor (FA)‐based counterparts.^10^ The superior photovoltaic performance of NFA OSCs is believed to originate from not only the prominent structural and optical characteristics in NFAs but also the favorable morphological and charge dynamic properties in the blend, the latter of which are however still obscure and require in‐depth understanding.

In FA‐based OSCs, a large energy offset^11^ of lowest unoccupied molecular orbital (LUMO) levels between organic semiconductor donor (D) and acceptor (A) is generally needed to overcome the high exciton binding energy (*E*_B_) of donor—typically within 0.33–0.7 eV,^12^, ^13^ which becomes an inevitable origin of the energy loss.^14^ Different from FA analogs, owing to the light absorption of both D and A components in NFA‐based systems, there exist two possible charge transfer channels—not only electron transfer from D to A but also hole transfer from A to D via highest occupied molecular orbital (HOMO) energy levels. Numerous studies have indicated that high driving force seems unnecessary for efficient hole transfer in NFA systems. For example, in the benchmark NFA systems based on 3,9‐bis(2‐methylene‐(3‐(1,1‐dicyanomethylene)‐indanone)‐5,5,11,11‐tetrakis(4‐hexylphenyl)‐dithieno[2,3‐d:2′,3′‐d′]‐s‐indaceno[1,2‐b:5,6‐b′]dithiophene (ITIC) and its derivatives, small HOMO energy offsets ranging from 0.04 to 0.2 eV were confirmed as sufficient to drive hole transfer by the means of photoluminescence (PL) quenching efficiency, transient absorption (TA) spectra, and so on.^7^, ^8^, ^15^, ^16^, ^17^ We consider such a phenomenon is presumably stemmed from the unique A–D–A‐type structure of NFAs, which permits the electron/hole pairs to be partly separated within the molecules by forming charge transfer excitons between the neighboring units and thus leads to a reduction of *E*_B_. However, whether the electron transfer can occur efficiently under a comparatively small LUMO offset in NFA‐based systems remains to be answered.

Despite the favorable energy level alignment of NFAs in OSCs, one major limiting factor is their high energetic loss caused by severe charge recombination induced by the non‐ideal film morphology. For instance, Wang and coworkers found that the charge recombination loss in the ITIC‐based OSCs was considerably worse than that in FA‐based counterparts due to the poor phase‐separation morphology induced by well miscibility of polymer donor and ITIC.^18^ One feasible approach to solve the problem is then the construction of ternary blend OSCs by judiciously selecting three components and tuning their ratio that can synergistically optimize film morphology, enhance optical absorption, and promote charge transport, which has attracted increasing research interest.^19^, ^20^, ^21^, ^22^, ^23^, ^24^, ^25^, ^26^, ^27^, ^28^

This contribution aims to gain a better understanding of efficient charge transfer and address the issue of morphology‐induced charge recombination in NFA‐based OSCs. We first designed and synthesized the random terpolymer donor based on one benzodithiophene (BDT) donor moiety and two well‐chosen acceptor units of benzodithiophene‐4,8‐dione (BDD)^29^ and fluorine‐substituted benzotriazole (FTAZ).^30^ At a molar ratio of BDT:BDD:FTAZ = 2:1:1, the resulting terpolymer donor named as **PBBF11** exhibits complementary light absorption and very small HOMO/LUMO energy offsets with ITIC, the latter of which can still ensure efficient hole and electron transfer between them. The BHJ OSCs based on the **PBBF11**:ITIC blend yield the maximum PCE of 10.02%. However, the homogeneous film morphology caused by the well miscibility between **PBBF11** and ITIC in the blend would induce severe charge recombination. Therefore, PC_71_BM was added into the **PBBF11**:ITIC system as the third component to modulate film morphology and improve charge transport. As a consequence, the optimal ternary OSC based on the **PBBF11**:ITIC:PC_71_BM (1:1:0.3, wt%) blend delivers an outstanding PCE of 11.4%.

## 2 Results and Discussion

**Figure 1:**
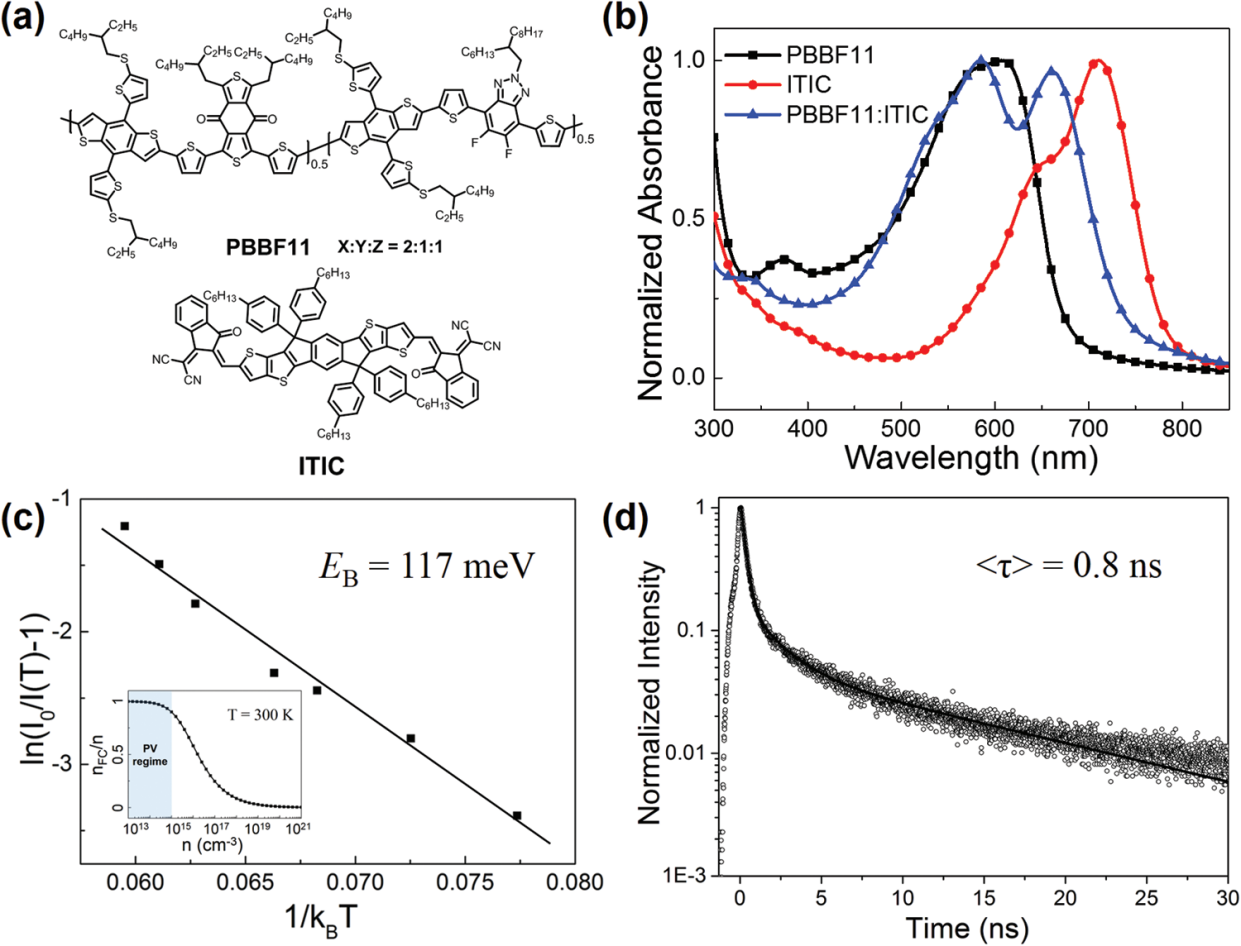
a) Molecular structures and b) UV–vis absorption spectra of **PBBF11** and ITIC neat, and **PBBF11**:ITIC blend films. c) Determination of *E*_B_ of ITIC by temperature‐dependent PL measurements with a linear fitting curve. Inset: A simulation of the free charge fraction over the total excitation density at 300 K under thermal equilibrium. The shaded area represents PV operating conditions (PV regime). d) TRPL decay of ITIC neat film.

Chemical structures of random terpolymer donor **PBBF11** and ITIC are depicted in **Figure** **1**a. **PBBF11** was synthesized via one‐pot Stille coupling reaction, and its synthetic route is shown in Figure S1 in the Supporting Information. As displayed in Figure 1b, the **PBBF11** neat film shows strong optical absorption ranging from 450 to 650 nm, which complements that of ITIC, and therefore the **PBBF11**:ITIC (1:1, wt%) blend film exhibits a broad coverage in the visible light region. The HOMO/LUMO energy levels of **PBBF11** are estimated to be −5.48/−3.66 eV while those of ITIC was reported as −5.51/−3.78 eV.^29^ It is noticed that the HOMO offset (Δ*E*_HOMO_) between **PBBF11** and ITIC is negligible as only 0.03 eV, which is however enough to achieve efficient hole transfer from ITIC to **PBBF11**. This is evidenced by the high PL quenching efficiencies over 90% for **PBBF11** to ITIC in the blend films (Figure S2, Supporting Information). Despite a small Δ*E*_HOMO_, it has been widely reported that ITIC‐based OSCs have demonstrated excellent device performance.^7^, ^8^, ^15^, ^16^, ^17^, ^31^ We therefore tend to believe ITIC should possess such unique features that differ from organic donors and FAs.

We for the first time characterized the exciton *E*_B_ of ITIC by measurements of temperature‐dependent PL intensity.^32^ The PL spectra of ITIC thin film were measured at low temperatures ranging from 140 to 200 K and the integrated PL intensities, *I*(*T*), were calculated. With increasing temperature, *I*(*T*) is decreased due to the thermal dissociation of excitons at higher temperature, and it can be expressed in Equation (1), 32:

(1) $$\frac{I\left(T_{0}\right)}{I\left(T\right)}=1+Ae^{\left(\frac{-E_{B}}{k_{b}T}\right)}$$

where *I*(*T*_0_) is the PL intensity at the lowest temperature, and *k*_B_ is the Boltzmann constant. The linear fitting of ln(*I*(*T*_0_)/*I*(*T*)−1) versus 1/(*k*_B_ *T*) is plotted in Figure 1c and, from the fitting slope, the *E*_B_ of ITIC is estimated to be ≈117 meV that is considerably low when comparing it to organic semiconductors which typically present an *E*_B_ of 0.2−0.4 eV.^33^ In this case, the photogenerated excitons might be intra‐charge transfer excitons along the neighboring D−A units rather than the Frenkel‐type excitons. Note that free charges and weak coupled excitons coexist under thermodynamic equilibrium with a stable molar ratio which resembles the ion–electron balance in a hot plasma expressed by using the Saha–Langmuir equation where the fraction of free charges over the total density of excitation, *x*, can be expressed in Equation (2), 34:

(2) $$\frac{x^{2}}{1-x}=\frac{1}{n}\;{\left(\frac{2\pi mk_{B}T}{h^{2}}\right)}^{1.5}e^{-\frac{E_{B}}{k_{B}T}}$$

where *E*_B_ is the exciton binding energy, *m* is the reduced mass of the exciton (approximated to 0.15 *m*_e_), *h* is Planck's constant, *T* is temperature, and *n* is the total density of excitation, *n* = *n*_FC_ + *n*_exc_. As shown in the inset of Figure 1c, most of photogenerated excitons in ITIC are separated into free charge carriers at room temperature (300 K) under photovoltaic (PV) operating conditions when *n* is ranging from 10^13^ to 10^15^ cm^−3^.^34^ Such a large fraction of free charge carriers in ITIC might account for why efficient hole transfer from ITIC to donor can occur even under a narrow Δ*E*_HOMO_. Therefore, the development of the donor with a similar HOMO level of ITIC is a feasible approach to maximize the open‐circuit voltage (*V*_OC_) without sacrificing the short‐circuit current density (*J*_SC_). Time‐resolved PL (TRPL) spectroscopic analysis was further conducted to unravel the detailed charge carrier dynamics of ITIC. As shown in Figure 1d, ITIC presents a longer PL lifetime of ≈0.8 ns than that of the benchmark P3HT (≈0.4 ns^35^). Then, the diffusion length (*L*_D_) of ITIC was estimated by using a 1D exciton diffusion equation as expressed in Equation (3), 36:

(3) $$L_{D}=\sqrt{D\tau }$$

where *D* is the diffusion coefficient and τ is the average PL lifetime in thin film, and *D* can be further calculated in Equation (4), 36:

(4) $$D\;=\sqrt{\frac{\mu k_{B}T}{e}}$$

where μ is the charge mobility and *e* is the electric quantity of elementary charge. Consequently, ITIC affords an *L*_D_ of ≈14.4 nm, which is larger than that of P3HT (*L*_D_ = ≈10.2 nm), indicating there are more chances for excitons to diffuse to the D/A interfaces which might enhance the toleration of blend film morphology on device performance in ITIC‐based systems.

**Figure 2:**
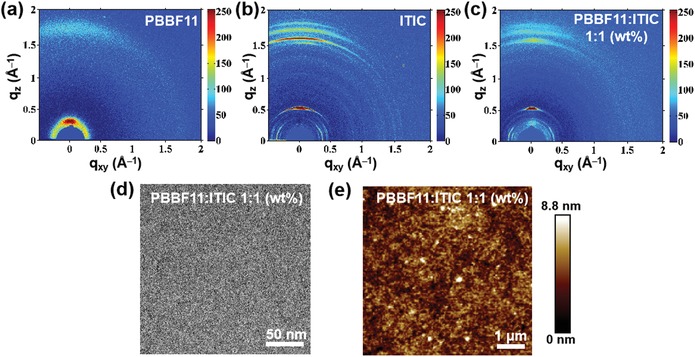
GIWAXS images of a) **PBBF11** and b) ITIC neat film, and c) **PBBF11**:ITIC (1:1, wt%) binary blend film. d) TEM and e) AFM images of **PBBF11**:ITIC (1:1, wt%) binary blend film.

Grazing‐incidence wide‐angle X‐ray scattering (GIWAXS) measurements were then conducted to evaluate the crystallinity properties of neat **PBBF11** and ITIC in solid state and their respective π‐stacking structures in the blend film. As shown in **Figure** **2**a, **PBBF11** exhibits a moderate (100) lamellar reflection peak located at 0.28 Å^−1^ along with a weak (010) π–π stacking peak at 1.73 Å^−1^ along the out‐of‐plane direction, which reflects its amorphous nature. On the contrary, ITIC displays two distinct (100) and (010) peaks at 0.52 and 1.62 Å^−1^ along out‐of‐plane direction, respectively, indicative of its relatively higher crystalline property than **PBBF11** (Figure 2b). Additionally, the ITIC neat film shows multiple signals along both in‐plane and out‐of‐plane directions, which are presumably attributed to various ITIC crystalline phases. In the **PBBF11**:ITIC blend film (Figure 2c), the signal intensities of ITIC are remarkably lower than that of ITIC neat film. This suggests that **PBBF11** is readily miscible with ITIC due to their similar chemical structures. Concurrently, the phase‐separated morphology and surface topography of the blend film were examined by transmission electron microscope (TEM) and atomic force microscope (AFM) techniques, respectively. A homogeneous blend film morphology is observed in Figure 2d, indicating the good miscibility of **PBBF11** and ITIC, which can also be confirmed from its low root‐mean‐square (RMS) surface roughness of 0.91 nm as shown in Figure 2e.

**Figure 3:**
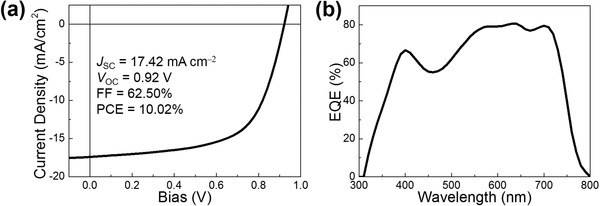
a) *J*–*V* curve and b) EQE spectrum of the **PBBF11**:ITIC‐based optimal OSC.

The additive‐ and annealing‐free BHJ OSCs were fabricated with an inverted configuration of ITO/ZnO/**PBBF11**:ITIC (1:1, wt%)/MoO_3_/Al and the resulting current density–voltage (*J*–*V*) curve is displayed in **Figure** **3**a. An impressive PCE of 10.02% was achieved with *J*_SC_ = 17.42 mA cm^−2^, *V*_OC_ = 0.92 V, and a fill factor (FF) of 62.50%. The current integrated from the external quantum efficiency (EQE) spectrum (Figure 3b) is 17.1 mA cm^−2^, which is about the same as *J*_SC_. High photocurrent is originated from the strong and complementary optical absorption of **PBBF11** and ITIC (Figure 1b) and efficient charge transfer between them as evidenced from the PL results (Figure S2, Supporting Information). Meanwhile, high *V*_OC_ is obtained because of the small Δ*E*_HOMO_ between **PBBF11** and ITIC.

**Figure 4:**
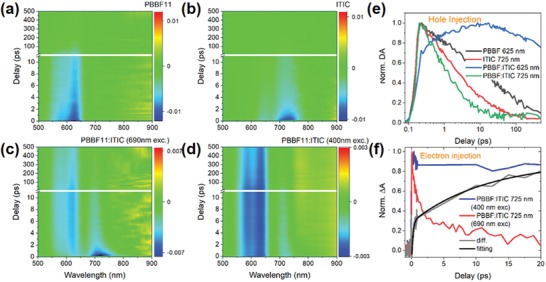
TA spectrograms of a) **PBBF11** and b) ITIC both excited at 400 nm, **PBBF11**:ITIC blend excited at c) 700 nm and d) 400 nm, respectively. TA kinetics of the samples illustrating e) hole transfer from ITIC to **PBBF11** and f) electron transfer from **PBBF11** to ITIC.

To unveil the detailed pathways of charge carrier transportation and recombination, ultrafast TA spectroscopy was utilized for mechanistic studies. The first factor to be confirmed is hole transfer from ITIC to **PBBF11**, which can be identified from the comparison of TA spectrograms among neat **PBBF11** (**Figure** **4**a), ITIC (Figure 4b), and **PBBF11**:ITIC blend (Figure 4c). After excitation above the *E*_g_s of both **PBBF11** and ITIC, the characteristic ground state bleaches (GSB) can be observed for both **PBBF11** (620 nm) and ITIC (720 nm) representing the population of the HOMO and LUMO energy levels as shown in Figure 4a,b, respectively. In the **PBBF11**:ITIC blend film (Figure 4c), the GSB of the **PBBF11** still appears when only ITIC is expected to be excited using 700 nm excitation. As the optical *E*_g_ of ITIC is comparatively narrower than that of **PBBF11** (as shown in Figure 1a), the shortened GSB recovery of **PBBF11** in the binary blend compared with that in neat **PBBF11** can be attributed to the efficient hole transfer from the HOMO energy level of ITIC to that of **PBBF11**.^16^ Such fast hole transfer can be further clarified from the extracted kinetics at the maximum bleach in Figure 4e where the TA kinetics in the blend exhibit faster decay for ITIC GSB (green curve) and slower rising for **PBBF11** GSB (blue curve) compared to neat samples (red and black curves), respectively. The hole transfer time can be estimated to be 1 ps from the fitting of the rising of TA kinetics for **PBBF11**:ITIC (blue line). Such efficient hole transfer in spite of low Δ*E*_HOMO_ is consistent with the reported results for ITIC, which will significantly increase the *V*_OC_ of OSC devices.^7^, ^8^, ^15^, ^16^, ^17^, ^31^

Apart from hole transfer, electron transfer from the LUMO level of **PBBF11** to ITIC is also essential to the generation of photocurrent. Thus, we first compared the TA spectrograms of the **PBBF11**:ITIC blend excited at 700 and 400 nm shown in Figure 4c,d, respectively. The TA kinetics at the maximum ITIC GSB (725 nm) of both cases are extracted into Figure 4f. When pumped at 700 nm, only ITIC in the blend would be excited and the GSB decay at 700−750 nm only reflects the depopulation of photoexcited charges in ITIC. However, by 400 nm pumping, both ITIC and **PBBF11** would be excited and the GSB dynamics in ITIC would be additionally contributed by either electron transfer or energy transfer from **PBBF11** as they are both energetically favorable in the blend system. In this scenario, the decay of ITIC GSB (blue line in Figure 4f) is much slower. Next, we extracted the differential curve (gray curve in Figure 4f) between two decays with 700 and 400 nm excitation. It can be fitted by two exponential rising components with lifetimes of 0.2 and 11 ps, respectively. The ultrafast component (0.2 ps) should be attributed to the electron transfer process which is notably faster than hole transfer from ITIC to **PBBF11**. The slower component (11 ps) is hard to assign at this stage since both the energy transfer and the dissociation of the intermediate charge transfer state (CTS) state could occur within this timescale in the blend system. However, efficient electron transfer from **PBBF11** to ITIC within 10 ps can still be qualitatively confirmed. In summary, we proved both electron and hole transfer in ITIC‐based blend film can be extremely efficient regardless of low driving force at both HOMO and LUMO energy levels.

**Figure 5:**
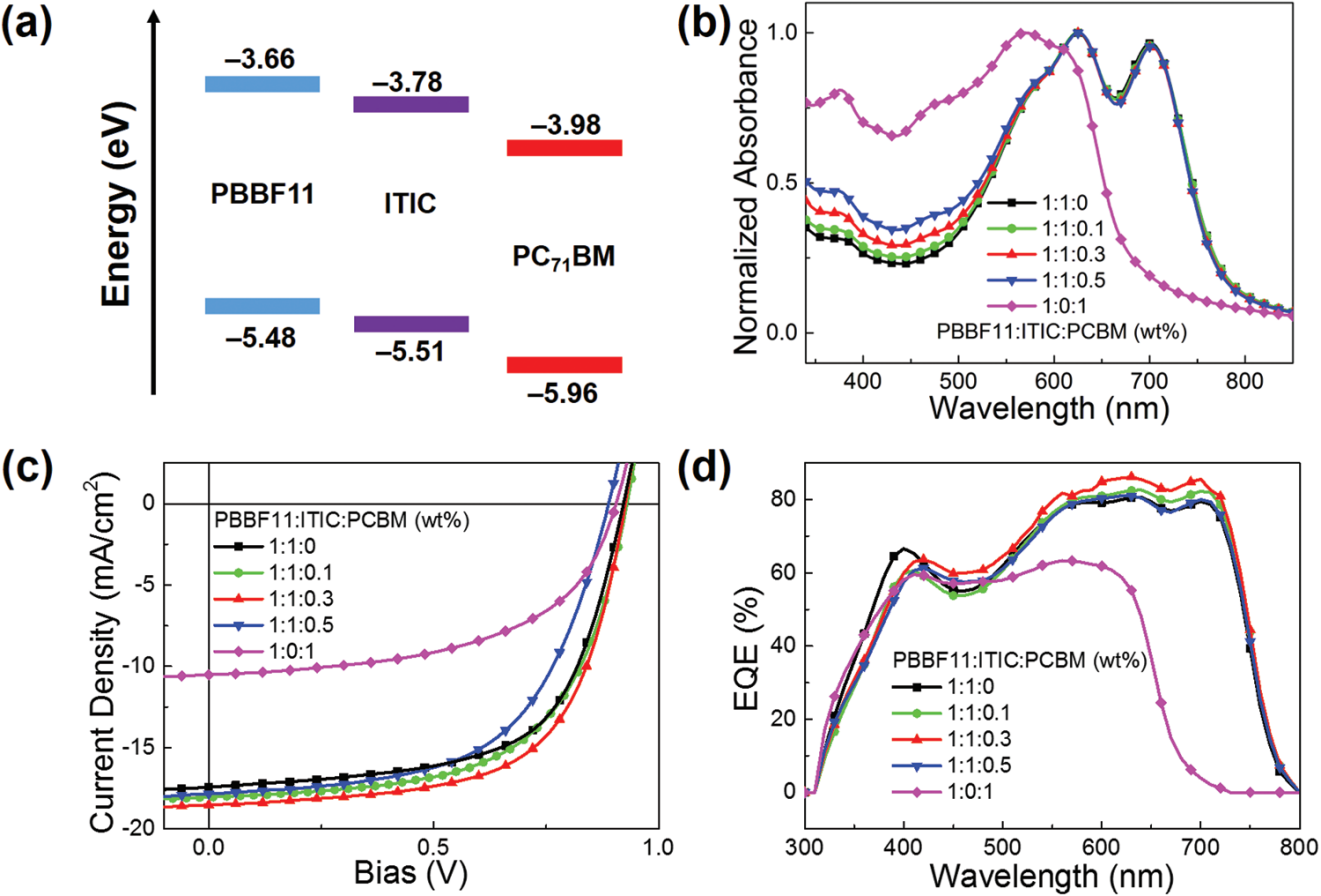
a) Energy level diagrams of **PBBF11**, ITIC, and PC_71_BM, respectively. b) UV–vis absorption spectra of the blend with different PC_71_BM ratios. c) *J*–*V* curves and d) EQE spectra of the optimal ternary OSCs.

Despite superior charge separation in the above **PBBF11**:ITIC blend film, the lifetime of separated charges in either donor or acceptor components is still ≈1−2 ns as evidenced by the TA decay at **PBBF11** GSB (625 nm) after ITIC is excited at 700 nm (Figure 4e, blue curve). This indicates that the interfacial geminate recombination still exists hindering charge transportation, which would be stemmed from the well miscibility between **PBBF11** and ITIC. In this respect, we set out to employ a third component to regulate the blend film morphology and further separate the electrons and holes from the D/A interfaces to retard the geminate recombination. PC_71_BM, which possesses strong electron‐withdrawing capability and lower LUMO energy level^24^ than ITIC (**Figure** **5**a), appears to be a suitable candidate. It can accept the electrons from both **PBBF11** and ITIC while the holes remain in **PBBF11**, thereby preventing the electrons and holes to be recombined at the interfaces of **PBBF11** and ITIC. Figure 5b shows the light absorption spectra of **PBBF11**:ITIC:PC_71_BM ternary blend films with various PC_71_BM loading ratios. It can be seen that PC_71_BM contributes little to light absorption due to its weak visible light harvesting characteristics and low addition amount.

Ternary **PBBF11**:ITIC:PC_71_BM and binary **PBBF11**:PC_71_BM OSCs were fabricated and compared with the same device structure and processing conditions as the **PBBF11**:ITIC‐based cells. Their *J*–*V* curves and EQE spectra are displayed in Figure 5c,d, respectively, and the corresponding photovoltaic parameters are collected in **Table** **1**. The *J*_SC_ deviations between *J*–*V* curves and EQE spectra are very low. Note that the ternary devices exhibited a bit lower EQE values in short wavelength regions than those of the binary PBBF11:ITIC devices, which might be ascribed to both the weak optical absorption of PCBM and the blend film morphology variation caused by the PCBM addition. Compared to the **PBBF11**:ITIC (1:1, wt%) binary device, the ternary systems based on **PBBF11**:ITIC:PC_71_BM (1:1:0.1 and 1:1:0.3, wt%) exhibit a steady increase of *J*_SC_, giving rise to prominent PCE enhancements. Note that the slightly enhanced optical absorption in ternary blends may not be the main contributor for the *J*_SC_ improvements (Figure 5b). Instead, the simultaneously boosted *J*_SC_ is believed to originate mainly from morphological and dynamic reasons induced by fullerene addition. When further increasing the weight ratio of PC_71_BM to 1:1:0.5, the device performance however begins to degrade with simultaneously reduced *J*_SC_, *V*_OC_, and FF. As a result, the optimal ternary blend OSCs based on **PBBF11**:ITIC:PC_71_BM (1:1:0.3, wt%) yield an outstanding PCE of 11.40% with *J*_SC_ = 19.17 mA cm^−2^, *V*_OC_ = 0.92 V, and FF = 64.65%.

**Table 1:** Summary of photovoltaic parameters of **PBBF11**:ITIC:PC_71_BM‐based devices under simulated light irradiation of 100 mW cm^−2^

Blend ratio [w/w/w]	*J*_SC_ a) [mA cm^−2^]	*J*_SC_ b) [mA cm^−2^]	*V*_OC_ a) [V]	FFa) [%]	PCEa) [%]
1:1:0	17.58 ± 0.42 (17.42)	17.1	0.91 ± 0.01 (0.92)	61.90 ± 0.59 (62.50)	9.94 ± 0.10 (10.02)
1:1:0.1	18.20 ± 0.24 (18.04)	17.3	0.91 ± 0.02 (0.92)	60.81 ± 1.31 (60.49)	10.07 ± 0.14 (10.15)
1:1:0.3	19.14 ± 0.77 (19.17)	18.0	0.92 ± 0.01 (0.92)	61.51 ± 1.94 (64.65)	10.88 ± 0.29 (11.40)
1:1:0.5	17.44 ± 0.37 (17.83)	17.1	0.90 ± 0.01 (0.90)	58.09 ± 0.56 (58.02)	9.01 ± 0.13 (9.21)
1:0:1	10.72 ± 0.17 (10.51)	10.2	0.90 ± 0.01 (0.90)	51.49 ± 2.40 (54.65)	4.97 ± 0.19 (5.17)

^a)^ The average values of photovoltaic parameters were obtained from at least five devices and the values in parentheses are the highest values

^b)^ The *J*_SC_ values were calculated from the EQE results.

**Figure 6:**
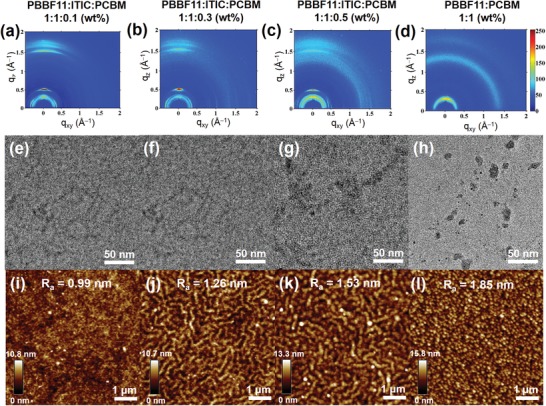
GIWAXS, TEM, and corresponding AFM images of ternary **PBBF11**:ITIC:PC_71_BM blends at a weight ratio of a,e,i) 1:1:0.1, b,f,j) 1:1:0.3, c,g,k) 1:1:0.5, and d,h,l) binary **PBBF11**:PC_71_BM (1:1, wt%) blend.

To reveal the morphological function of PC_71_BM, GIWAXS, TEM, and AFM measurements of ternary blend films were successively conducted. The GIWAXS diffractograms of **PBBF11**:ITIC:PC_71_BM‐based blend films are presented in **Figure** **6**a–d. The characteristic diffraction ring of PC_71_BM (at ≈1.40 Å^−1^; see Figure S3, Supporting Information) becomes gradually distinct with an increase of PC_71_BM addition amount, indicative of the formation of individual PC_71_BM aggregates. Additionally, it is interesting to note that in the **PBBF11**:ITIC:PC_71_BM (1:1:0.3, wt%) ternary blend, several extra diffraction peaks appear along the in‐plane direction, which could be ascribed to the enhanced crystallinity of ITIC, implying that an addition of PC_71_BM might further induce the ITIC crystallization. TEM and AFM images offer more morphological evidences as shown in Figure 6e–l. In the ternary blends, an introduction of PC_71_BM is found to afford additional PC_71_BM phases as seen by the existence of black dots (≈10 nm) in the TEM images (Figure 6e–g) with various fullerene loading ratios, in which an increasing PC_71_BM ratio leads to rougher surface (Figure 6i–k). By varying the amount of PC_71_BM aggregates, the blend film morphology can be finely regulated, and thus a remarkable enhancement of device performance is achieved with an appropriate PC_71_BM ratio (30 wt% vs ITIC). However, the **PBBF11**:PC_71_BM binary blend shows distinctive PC_71_BM aggregates (Figure 6h) with the highest RMS roughness of 1.85 nm (Figure 6l), which would impede charge separation at D/A interfaces.

**Figure 7:**
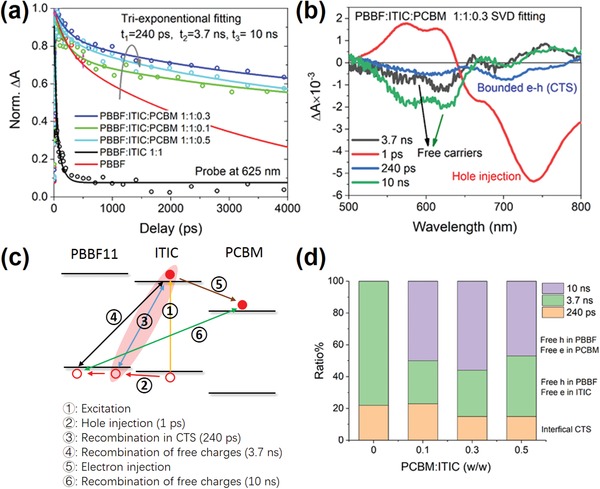
a) TA kinetics (open dots) of the blends with various PC_71_BM weight ratios along with their global fitting (solid lines) of tri‐exponential functions, b) SVD fitting of the TA spectrogram of optimal blend, c) schematics of the charge recombination pathways, and d) evolution of the amplitudes among three decay components in the blend samples.

To comprehend the difference of charge carrier transportation and recombination dynamics between **PBBF11**:ITIC:PC_71_BM and **PBBF11**:ITIC‐based systems, ultrafast TA spectroscopy studies were then performed as shown in **Figure** **7**. When PC_71_BM is added into the binary blend, the lifetime of injected holes in **PBBF11** becomes longer as shown in the GSB of **PBBF11** at 620 nm in Figure 7a. The underlying mechanism can also be analyzed by the singular value decomposition (SVD) fitting of TA spectra in the ternary blends as summarized in Figure 7b. During the SVD fitting, we first obtained the rate constants of each component from the conventional multi‐exponential fitting of all the main bleaching bands as the initial parameters. It should also be noted that we have minimized the excitation intensity down to the fluence of 1 × 10^12^ ph cm^−2^ pulse^−1^. Thus, the contribution of all high‐order nongeminate recombination within ITIC or **PBBF11** should be neglected in our case. Besides the 1 ps component denoted for hole transfer, three more components with lifetime of 240 ps (blue), 3.7 ns (black), and 10 ns (green) can also be extracted. Among them, the blue component of 240 ps consists of negative signals at the positions of GSB for both ITIC and **PBBF11**. Such a component can also be extracted in the binary **PBBF11**:ITIC blend (Figure S4a, Supporting Information) with similar spectral feature and lifetime after hole transfer occurs from ITIC to **PBBF11**. This means it should reflect a status with electron residing in ITIC and hole locating in **PBBF11** concurrently. In addition, we notice the spectral feature of the negative bleach around 600 nm in this 240 ps component is different from that in neat **PBBF11** film (Figure S4b, Supporting Information). This suggests the holes are not directly injected into the HOMO energy level of **PBBF11**, which is a signature feature of the intermediate CTS. The other two slow components with lifetimes of 3.7 and 10 ns share the same spectral feature of the TA spectrum for neat **PBBF11** (Figure S4b, Supporting Information), which can be thus attributed to the injected holes on the HOMO energy level of **PBBF11**. We also note that the 3.7 ns component can also be extracted in the SVD fitting of the binary **PBBF11**:ITIC blend (Figure S4a, Supporting Information), while the 10 ns component is not observed. This means the former refers to the charge dynamics within **PBBF11** and ITIC whereas the long‐lived component should reflect that induced by the addition of PC_71_BM. Based on the above analysis, we propose all the photoexcited charge carrier recombination pathways in the ternary blend as schematically shown in Figure 7c. The ultrafast (1 ps) hole transfer to **PBBF11** directly from the photoexcitation in ITIC leads to the formation of the interfacial CTS with a dissociation time of 240 ps. After dissociation, the free charges in **PBBF11** and ITIC still have a chance to recombine at the interface within 3.7 ns. The role of PC_71_BM is then to extract the remaining electron carriers in ITIC after hole transfer to **PBBF11**, which reduces the possibility for them to recombine with hole carriers in **PBBF11** due to both the long distance between free electrons and holes and the lowered driving force for charge recombination. We believe these two factors account for prolonged charge lifetime and improved device efficiency afforded by PC_71_BM addition.

Despite the fact that a small addition of fullerene can boost the device performance of NFA‐based systems has been widely reported,^24^, ^25^, ^26^, ^27^, ^37^, ^38^, ^39^ the role FA plays in ternary NFA blends and devices, particularly in relation to the loading percent of fullerene, has yet to be systematically explored. We therefore investigated the influence of the PC_71_BM addition ratio on charge dynamics by TA studies. In order to simplify the analysis, we conducted the global fitting of the TA kinetics at the **PBBF11** bleach maximum (625 nm) for the blend films with various PC_71_BM ratios along with **PBBF11** neat film. The evolution of the corresponding amplitude for each component is summarized in Figure 7d. In binary **PBBF11**:ITIC, only recombination within the CTS (orange) and between interfacial free charges (green) can be found with the major contribution from the free charges (78%). When 10 wt% PC_71_BM (relative to ITIC) is added, the recombination of the most dissociated charges from CTS between **PBBF11** and ITIC is replaced by charge recombination between **PBBF11** and PC_71_BM (purple 50%) with the contribution of CTS unchanged. Further addition of PC_71_BM to 30 wt% increases charge recombination between **PBBF11** and PC_71_BM (56%) and reduces CTS (geminate) recombination (15%). When the ratio of PC_71_BM reaches 50 wt%, the interfacial charge recombination between **PBBF11** and ITIC becomes pronounced again (38%). In short, inserting PC_71_BM into the binary blends initially retards the charge recombination paths between **PBBF11** and ITIC; yet an increasing addition of PC_71_BM would then reverse such an effect.

**Figure 8:**
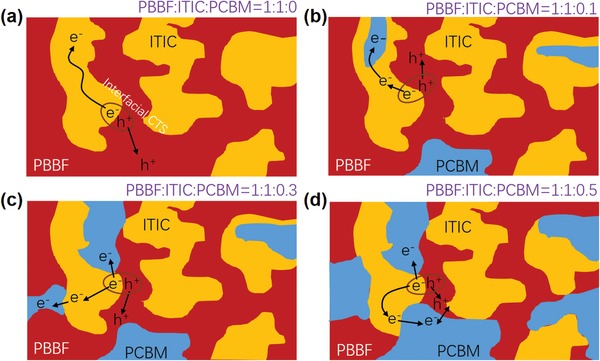
Schematics of charge transportation pathways in a) **PBBF11**:ITIC (1:1, wt%) binary, b) **PBBF11**:ITIC:PC_71_BM (1:1:0.1, wt%) ternary, c) **PBBF11**:ITIC:PC_71_BM (1:1:0.3, wt%) ternary, and d) **PBBF11**:ITIC:PC_71_BM (1:1:0.5, wt%) ternary blends.

According to the literature, the influence of the D/A ratio in the BHJ blends of OSCs on charge transport dynamics is mainly originated from the microscopic film morphologies.^40^ Although further structural characterization is needed to reveal such a correlation, we can still propose one of the most reasonable morphologic models as illustrated in **Figure** **8** to interpret the obtained photophysics. In the binary blend, both ITIC and amorphous‐like **PBBF11** phases are homogeneously mixed, which ensures the efficient interfacial charge separation (Figure 8a). Then, a small amount of added PC_71_BM is more likely embedded merely within either ITIC or **PBBF11** domains where only electron transfer from ITIC to PC_71_BM is energetically favorable and thus feasible (Figure 8b). When the ratio of PC_71_BM is increased to 30 wt% versus ITIC, the chance is that the PC_71_BM phase can reach the interfaces of ITIC and **PBBF11** (Figure 8c). In this scenario, electron transfer should occur not only for free charge carriers but also for interfacial CTS. In other words, the separation of charge carries would be maximized. Overaddition of PC_71_BM, however, would induce extra recombination pathways for the electrons in PC_71_BM and the holes in **PBBF11** if they are encountered (Figure 8d).

## 3 Conclusions

In conclusion, we have designed and synthesized a terpolymer donor of **PBBF11** which affords complementary absorption regions and small HOMO/LUMO offsets with ITIC NFA. Efficient electron and in particular hole transfer between them are verified by both PL and TA studies along with the small *E*_B_ of ITIC. Consequently, **PBBF11**:ITIC‐based OSCs yield a high PCE of 10.02%. Severe charge recombination is however found and therefore we introduce a small amount of PC_71_BM to optimize the blend film morphology and effectively extract the photogenerated electrons. At the optimal addition ratio of PC_71_BM, the fullerene aggregates can connect both ITIC domains and **PBBF11**:ITIC interfaces in the blend, which ensures the swift separation of CTS and the efficient extraction of free electrons, thereby largely suppressing charge recombination. As a result, the optimized **PBBF11**:ITIC:PC_71_BM (1:1:0.3, w/w/w) ternary OSCs achieved the best PCE of 11.4% with *V*_OC_ = 0.92 V, *J*_SC_ = 19.17 mA cm^−2^, and FF = 64.65%. Therefore, this work not only fundamentally reveals the reasons why efficient hole transfer occurs under negligible HOMO offset in ITIC‐based OSCs but also manifests the great promise of exploiting FA into NFAs‐based OSCs.

## Conflict of Interest

The authors declare no conflict of interest.

## Supporting information

Supplementary
