# Supplementary material for: Unveiling Excitonic Dynamics in High‐Efficiency Nonfullerene Organic Solar Cells to Direct Morphological Optimization for Suppressing Charge Recombination
Source: Adv Sci (Weinh). 2019 Feb 19;6(8):1802103. doi: 10.1002/advs.201802103 (PMC6468965; doi:10.1002/advs.201802103)
Supplement: Supplementary file 1 — Supplementary [file ADVS-6-1802103-s001.pdf]

Copyright WILEY-VCH Verlag GmbH & Co. KGaA, 69469 Weinheim, Germany, 2019.

ADVANCED SCIENCE
Open Access

Supporting Information

for *Adv. Sci.,* DOI: 10.1002/advs.201802103

Unveiling Excitonic Dynamics in High-Efficiency Nonfullerene Organic Solar Cells to Direct Morphological Optimization for Suppressing Charge Recombination

Xiaoyu Liu, Yajie Yan, Alireza Honarfar, Yao Yao, Kaibo Zheng, and Ziqi Liang**

Supporting Information

Unveiling Excitonic Dynamics in High-Efficiency Nonfullerene Organic Solar Cells to Direct Morphological Optimization for Suppressing Charge Recombination

Xiaoyu Liu,[1] Yajie Yan,[1] Alireza Honarfar,[3] Yao Yao,[4] Kaibo Zheng,[2,3*] and Ziqi Liang[1*]

[*][1]Prof. Z. Liang, X. Liu, Y. Yan
Department of Materials Science
Fudan University
Shanghai 200433, China
Email: zqliang@fudan.edu.cn

[*][2]Dr. K. Zheng
Department of Chemistry
Technical University of Denmark
DK-2800 Kongens Lyngby, Denmark
Email: kzheng@kemi.dtu.dk

[3]Dr. K. Zheng, A. Honarfar
Department of Chemical Physics and NanoLund
Lund University
P.O. Box 124, 22100 Lund, Sweden

[4]Dr. Y. Yao
Department of Physics and State Key Laboratory of Luminescent Materials and Devices
South China University of Technology
Guangzhou 510640, China

Experimental Section

Materials: (4,8-bis(5-((2-ethylhexyl)thio)thiophen-2-yl)benzo[1,2-b:4,5-b']dithiophene-2,6-diyl)bis(trimethylstannane) (Me_3Sn–BDT–$SnMe_3$), 1,3-bis(5-bromothiophen-2-yl)-5,7-bis(2-ethylhexyl)-4H,8H-benzo[1,2-c:4,5-c']dithiophene-4,8-dione (Br–BDD–Br) and 4,7-bis(5-bromothiophen-2-yl)-5,6-difluoro-2-(2-hexyldecyl)-2H-benzo[d][1,2,3]triazole (Br–FTAZ–Br) were purchased from Derthon Optoelectronic Materials Science & Technology Co., Ltd. $Pd(PPh_3)_4$ was purchased from J&K Scientific, Ltd. (China). $PC_{71}BM$ was purchased from Nano-C, Inc. ITIC was purchased from Solarmer, Ltd. (China) Other chemical reagents were purchased from J&K Scientific, Ltd. (China), Sigma-Aldrich, Ltd. (China) and used as received, except toluene was redistilled before utilization.

Synthesis for Terpolymer PBBF11: Me_3Sn–BDT–$SnMe_3$ (200 mg, 0.2064 mmol), Br–BDD–Br (79.1 mg, 0.1032 mmol), Br–FTAZ–Br (72.4 mg, 0.1032 mmol) were dissolved in 10 mL of dry toluene, and the solution was flushed with nitrogen for 10 min. Then, $Pd(PPh_3)_4$ (11.9 mg, 0.0103 mmol) was added to the solution and the mixture was flushed again with nitrogen for 10 min. The copolymerization was carried out under nitrogen atmosphere at 120 °C for 20 h. When the reaction system was cooled down to room temperature, the obtained mixture were slowly dropped into methanol (250 mL). Subsequently, collected by filtration, the precipitate was further purified by Soxhlet extraction with methanol, acetone, hexane, and chloroform successively. The polymer was recovered as solid from the chloroform solution by again precipitation from methanol. The attained purple gold solid was dried under vacuum for 24 h and then transferred to the nitrogen-filled glovebox. For **PBBF11**, ^{1}H NMR (500 MHz, $CDCl_3$): δ (ppm) 13.52 (s, 1H), 12.48 (s, 1H), 10.12 (s, 3H), 8.79 (s, 4H), 7.45 (s, 1H), 7.33 (s, 1H), 6.07 (s, 1H), 3.99 (s, 1H), 3.78 (s, 1H), 3.70 (s, 1H), 3.36 (s, 1H), 3.04 (s, 172H), 2.80 (s, 4H), 2.48 (s, 4H), 1.66 (s, 48H), 1.59 (s, 12H), 1.51 (s, 12H). GPC: M_n = 60.2 kDa, M_w = 191.8 kDa, M_w/M_n (PDI) = 3.2.

Characterization: The ^{1}H NMR spectrum was acquired at room temperature on Bruker DMX-500 spectrometer with a solvent of $CDCl_3$ and an internal standard of tetramethylsilane (TMS). The Gel permeation chromatography (GPC) measurement was carried on to determine the number-average molecular weights (M_n), weight-average molecular weights (M_w), and polydispersity index (PDI, M_w/M_n) with polystyrene as reference standard and THF as an eluent at room temperature. Optical absorption and photoluminescence spectra of thin film samples spin-casted from chlorobenzene solution were recorded on Agilent 8453 UV–vis spectrophotometer and a Horiba FluoroMax-4 spectrofluorometer, respectively. Cyclic voltammetry (CV) measurement was done on the Model 660E Series Potentiostat/Galvanostat (CH Instruments, Inc.). Grazing incident wide-angle X-ray scattering (GIWAXS) measurements were conducted at the beamline BL14B1 with an energy of 10 kV, a wavelength of 1.2398 nm and a pixel size of 73.2419 μm in Shanghai Synchrotron Radiation Facility (SSRF). The detailed information about beamline BL14B1 can be found in Ref. [S1]. The variable q_{xy} and q_z are the components of the scattering vector parallel and perpendicular to the substrate, respectively. q can be calculated from the equation of $q = (4\pi/\lambda)\sin\theta$, where λ is the incident radiation wavelength and θ is a half of the scattering angle. Thin film samples were fabricated by spin-coating the solutions in chlorobenzene on the silicon wafers. High-resolution transmission electron microscopy (TEM) imaging was performed on Tecnai G2 F20 S-Twin microscope at an accelerating voltage of 200 kV. Bruker Dimension Edge atomic force microscope (AFM) in the tapping mode was utilized to image blend film topographies. The height images were gained at a scan rate of 1 Hz with a resolution of 512 × 512 pixels using a silicon etched tip, which has a resonance frequency of ≈300 kHz and a spring constant of ≈40 N m^{-1}. Transient absorption (TA) experiments were performed by using a femtosecond pump-probe setup in nitrogen atmosphere. Laser pulses (800 nm, 80 fs pulse length, 1 kHz repetition rate) were generated by a regenerative amplifier (Spitfire XP Pro) seeded by a

femtosecond oscillator (Mai Tai SP, both Spectra Physics). The pump pulses at 400 nm were generated by a BBO crystal as a second harmonic of the laser. The pump pulses at 700 nm were generated by an optical parametric amplifier (Topas, Light Conversion). The used excitation photon fluxes are 1×10^{12} photons/cm2/pulse. For the probe, we used the super-continuum S3 generation from a thin CaF2 plate. The mutual polarization between pump and probe beams was set to the magic angle (54.7°) by placing a Berek compensator in the pump beam. The probe pulse and the reference pulse were dispersed in a spectrograph and detected by a diode array (Pascher Instruments). In order to avoid photo-damage, the sample was moved to a fresh spot after each time delay point. Global SVD analysis was performed with the Glotaran software package (http://glotaran.org). These methods yield more accurate fits of rate constants because they treat the full data set as a whole. A simple sequential decay model with various components is chosen for every fitting. For TRPL measurement, time-correlated single-photon counting (TCSPC) device (PicoQuant) a pulsed diode laser with 100k repetition rate was used to excite the sample at 438 nm. The emitted photons were focused onto a fast avalanche photodiode (Micro Photon Device, SPAD). The excitation photon flux was also kept to be 1.0×10^{12} photons/cm^2/pulse.

Device Fabrication and Measurements: Patterned indium tin oxide (ITO) substrates were cleaned successively in an ultrasonic solvent bath of deionized water, acetone, and isopropyl alcohol for 20 min, respectively. Then, the ITO substrates were dried with heat gun and further treated in an ozone reactor for 20 min. Subsequently, the ZnO precursor solution was spin-coated onto the pre-cleaned ITO substrates at 5000 rpm for 30 s and annealed at 200 ºC for 30 min in air to form a ZnO layer of ~30 nm. After cooling to room temperature, the ZnO-coated substrates were transferred to a nitrogen-filled glovebox. Next, **PBBF11**:ITIC:$PC_{71}BM$ in chlorobenzene solution (total concentration: 20 mg/mL) with various weight ratio ranging from 1:1:0, 1:1:0.1, 1:1:0.3, 1:1:0.5 and 1:0:1, were spin-coated onto the ZnO-coated

substrates at 1800–2200 r.p.m. for 60 s. The thickness of the active layer was ~100 nm and the effective area of one cell was 0.04 cm^2. Finally, MoO_3 (10 nm) and Al (100 nm) layers were deposited by thermal evaporation under a pressure of <10^{-4} Pa at a rate of ~0.2 and ~1.0 Å/s, respectively. The J–V data were acquired from a Keithley 2400 source–meter unit. The light J–V curves were measured under light illumination with a Newport-Oriel (Sol3A Class AAA Solar Simulator, 94043A) AM 1.5G light source operating at an intensity of 100 mW cm^{-2}. The light intensity was calibrated by a certified Oriel reference cell (91150V) and verified with a NREL calibrated, filtered silicon diode (Hamamatsu, S1787-04). External quantum efficiency (EQE) spectra were measured on a commercial EQE set-up (QE-R, Enli Technology Co., Ltd). A calibrated silicon diode with a known spectral response was used as a reference. Hole and electron mobilities were attained by using the space charge limited current (SCLC) method, which were calculated with MOTT–Gurney equation:

$$J = \frac{9\varepsilon_0\varepsilon_r\mu V^2}{8L^2} \quad (1)$$

where J is the current density, ε_0 is the dielectric constant of empty space, ε_r is the relative dielectric constant of active layer materials which is taken to be 3 in the calculation, μ is the charge mobility, V is the internal voltage in the device, and L the thickness of the active layers. V can be calculated as $V = V_{appl} - V_{bi}$, where V_{appl} is the voltage applied to the devices, V_{bi} is the built-in voltage from the relative work function difference between the two electrodes.

Results and Discussion

Synthesis of **PBBF11**: Random terpolymer donor of **PBBF11** was designed and synthesized via one-pot Stille coupling reaction and its synthetic route is shown in Figure S1. **PBBF11** comprises one D moiety of benzodithiophene (BDT) and two A units of benzodithiophene-4,8-dione (BDD) and fluorine substituted benzotriazole (FTAZ) to

generate a D–A alternated backbone structure. The molar ratio of BDT, BDD and FTAZ is fixed to 2:1:1, in which the BDD and FTAZ segments are randomly inserted into the adjacent BDT units.

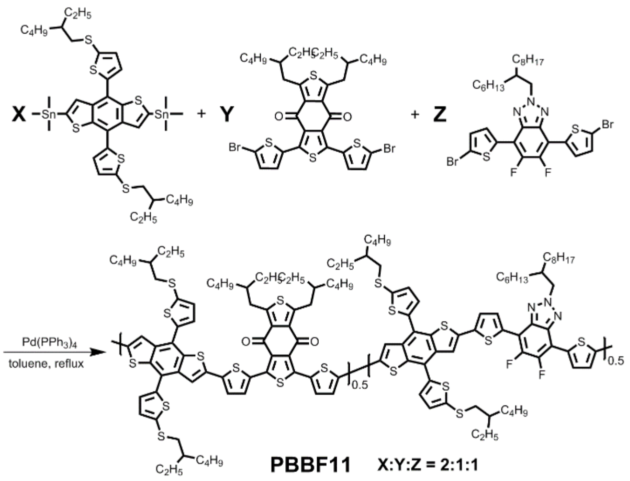

Figure S1. Synthetic route of **PBBF11** donor.

PL quenching: The photoluminescence (PL) quenching efficiencies (PL_{QE}) are calculated by equation:

$$PL_{QE} = 1 - PL_{blend}/PL_{neat} \tag{2}$$

where the PL_{neat} and PL_{blend} are referred to the integrated PL intensities of neat ITIC film, and **PBBF11**:ITIC:PC_{71}BM based blend films, respectively. As shown in Figure S2, all of the blends present high PL quenching efficiencies over 90%.

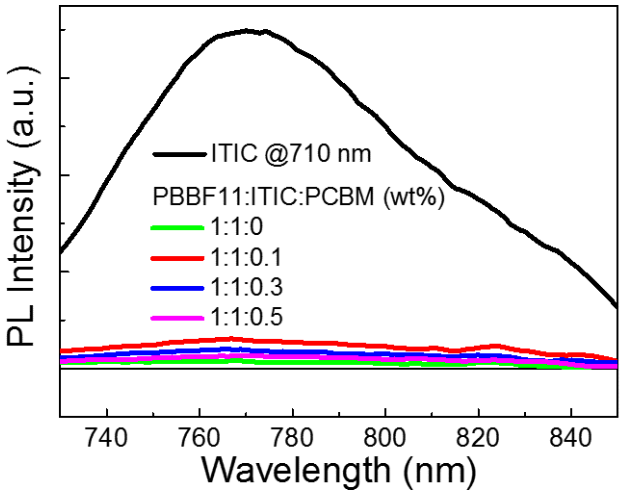

Figure S2. PL spectra of ITIC neat film and **PBBF11**:ITIC:$PC_{71}BM$ based blend films excited at 710 nm.

GIWAXS result of $PC_{71}BM$: As shown in Figure S3, the grazing-incidence wide-angle X-ray scattering (GIWAXS) pattern of $PC_{71}BM$ neat film exhibits a weak diffraction ring at ca. 1.40 $Å^{-1}$, which indicated its amorphous nature.

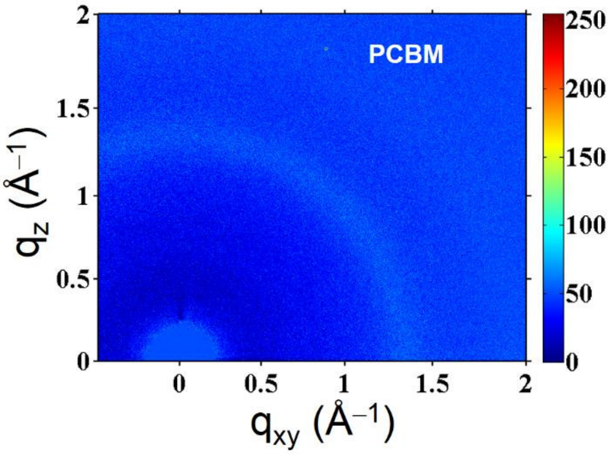

Figure S3. GIWAXS image of $PC_{71}BM$ neat film.

SVD fitting of the **PBBF11**:ITIC blend film: The singular value decomposition (SVD) fitting of **PBBF11**:ITIC is proceeded as shown in Figure S5a, from which three components of 3.7 ns, 240 ps and 1 ps are extracted. The 1 ps component refers to the hole transfer from ITIC to **PBBF11**. The 240 ps component reflects a status with electron residing in ITIC and hole locating in **PBBF11** concurrently. Also, the spectral feature of the negative bleach around 600 nm in this 240 ps component is different from that in neat **PBBF11** film (Figure S4b). This suggests the holes are not directly injected into the HOMO energy level of **PBBF11**, which is a signature feature of the intermediate charge transfer state (CTS). We also note that the 3.7 ns component can also be extracted in the SVD fitting of the binary **PBBF11**:ITIC blend (Figure S4a), while the 10 ns component which can be extracted in **PBBF11**:ITIC:$PC_{71}BM$ blend (Figure 7b) is not observed. This means the former refers to the

charge dynamics within **PBBF11** and ITIC whereas the long-lived component should reflect that induced by the addition of $PC_{71}BM$.

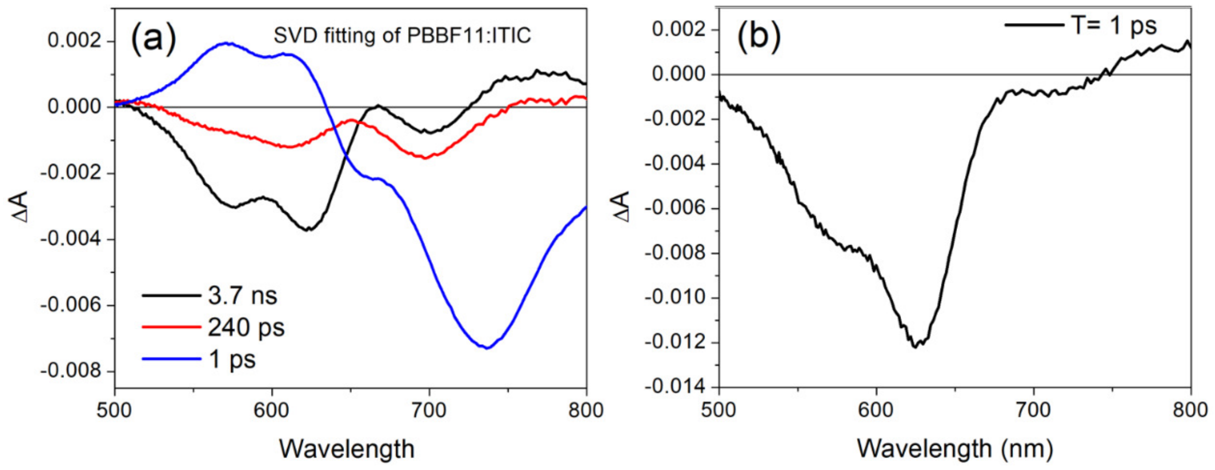

Figure S4. (**a**) SVD fitting components of **PBBF11**:ITIC blend film excited at 700 nm. (**b**) TA spectrum of **PBBF11** neat film at delay time of 1 ps excited at 400 nm.

Energy levels of **PBBF11**, ITIC and $PC_{71}BM$: The HOMO energy level of **PBBF11** was determined by cyclic voltammetry (CV) method. A conventional three-electrode system was adopted, including a fresh Au working electrode, a saturated Ag/AgCl as the reference electrode, and a platinum wire as the counter electrode in a nitrogen–saturated acetonitrile solution of 0.1 M tetrabutylammonium hexafluorophosphate (Bu_4NPF_6) electrolyte. By using a ferrocene/ferrocenium (Fc/Fc^+) redox couple (4.80 eV below the vacuum level) as an external standard, the HOMO energy level of **PBBF11** is estimated to be –5.48 eV from the oxidation potential. On the other hand, the optical bandgap (E_g^{opt}) of **PBBF11** can be calculated by its absorption edge at ~680 nm to be a medium bandgap value of ~1.82 eV, and its LUMO energy level can be calculated to be –3.66 eV according to $E_{LUMO} = E_{HOMO} + E_g^{opt}$. The energy levels of ITIC and $PC_{71}BM$ are obtained from the previous reports.[S2,S3]

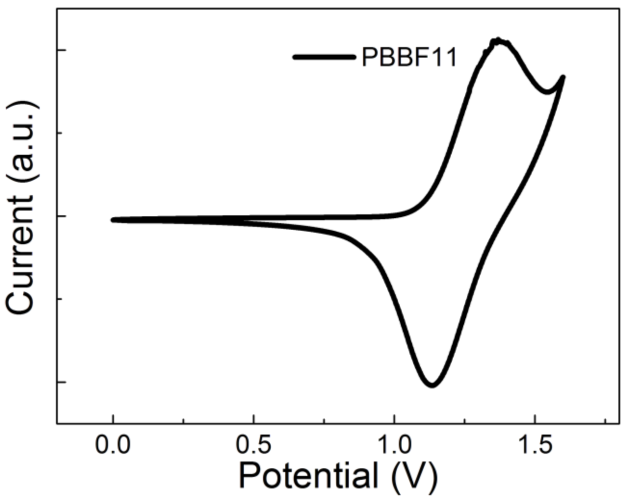

Figure S5. CV curve of **PBBF11**.

Transport physics in device: The photocurrent density versus the effective applied voltage (J_{ph}–V_{eff}) characteristics and the dependence of J_{SC} on light illumination intensities (P_{light}) were measured as shown in Figure S6.[S4] The obtained results are summarized in Table S1. Note that $J_{ph} = J_{light} - J_{dark}$ where J_{light} and J_{dark} refer to the current densities under illumination and in the dark, respectively, while $V_{eff} = V_0 - V_a$ where V_0 and V_a refers to the voltage when $J_{ph} = 0$ and the applied bias, respectively. At high V_{eff} (e.g., ≥3 V), all the photo-generated excitons are dissociated and collected by respective electrodes, and the saturation photocurrent density (J_{sat}) is only limited by the absorbed incident photons. Thus, J_{SC}/J_{sat} can be defined as the efficiency of charge extraction under short-circuit condition, which are calculated to be 89.1%, 90.4%, 93.0%, 90.1% and 86.3% in our **PBBF11**:ITIC:$PC_{71}BM$ devices with the component ratio of 1:1:0, 1:1:0.1, 1:1:0.3, 1:1:0.5 and 1:0:1 (wt%), respectively. These results indicate that ternary devices with an appropriate $PC_{71}BM$ loading percent exhibit more efficient charge dissociation and collection than the binary devices due to their favorable blend film morphologies. Furthermore, the dependence of J_{SC} on P_{light} was studied to probe the carrier recombination behaviors of the devices and then fitted with the equation of $J_{SC} \propto P_{light}^{\alpha}$.[S5] The obtained α values are 95.9%, 98.1%, 99.2%, 98.0% and

96.8% in **PBBF11**:ITIC:$PC_{71}BM$ (1:1:0, 1:1:0.1, 1:1:0.3, 1:1:0.5 and 1:0:1, wt%) based devices, respectively. Among them, the α value in 1:1:0.3 (wt%) based device is close to 1, indicating its negligible bimolecular recombination which contributes to higher photocurrent.

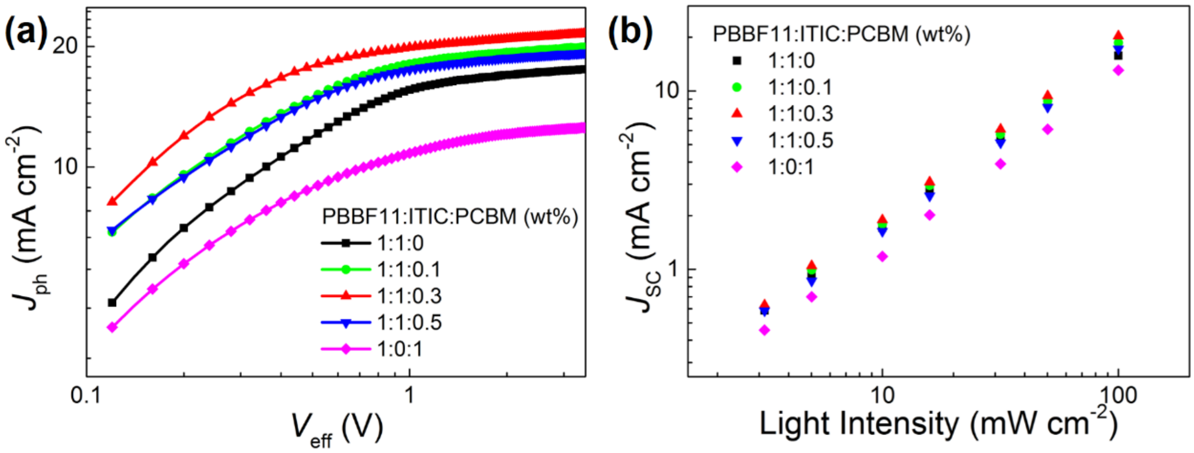

Figure S6. J_{ph}–V_{eff} and J_{SC}–P_{light} curves of the optimal OSCs based on **PBBF11**:ITIC:$PC_{71}BM$ blends. (**a**) J_{ph}–V_{eff} and (**b**) J_{SC}–P_{light} curves.

Charge mobilities: The space charge-limited current (SCLC) measurements with hole-only device of ITO/PEDOT:PSS/active layer/Au and electron-only device of ITO/ZnO/active layer/Ca/Al were conducted to calculated electron and hole mobilities in the devices, respectively. Dark J–V curves of the devices are fitted to the SCLC model, as shown in Figure S7. The calculated hole (μ_h) and electron (μ_e) mobilities along with μ_h/μ_e values are summarized in Table S1. When tuning the weight ratio of the **PBBF11**:ITIC:$PC_{71}BM$ based devices from 1:1:0, 1:1:0.1, 1:1:0.3, 1:1:0.5 to 1:0:1, μ_h decreases from 3.56, 2.75, 2.47, 1.67 down to 1.41 × 10^{-3} cm^2 V^{-1} s^{-1}. On the contrary, μ_e increases from 1.23, 2.28, 2.46, 2.96 up to 9.92 × 10^{-3} cm^2 V^{-1} s^{-1}, suggesting that $PC_{71}BM$ possesses better electron transporting property than ITIC. One plausible reason is that the bulky side chains located on the donor core of ITIC hinder charge transportation while $PC_{71}BM$ permits efficient electron transportation in three dimensions.[S6] consequently, the optimal ternary device (1:1:0.3, wt%)

exhibits the most balanced μ_h / μ_e value near the unity, which corresponds to the highest FF value.

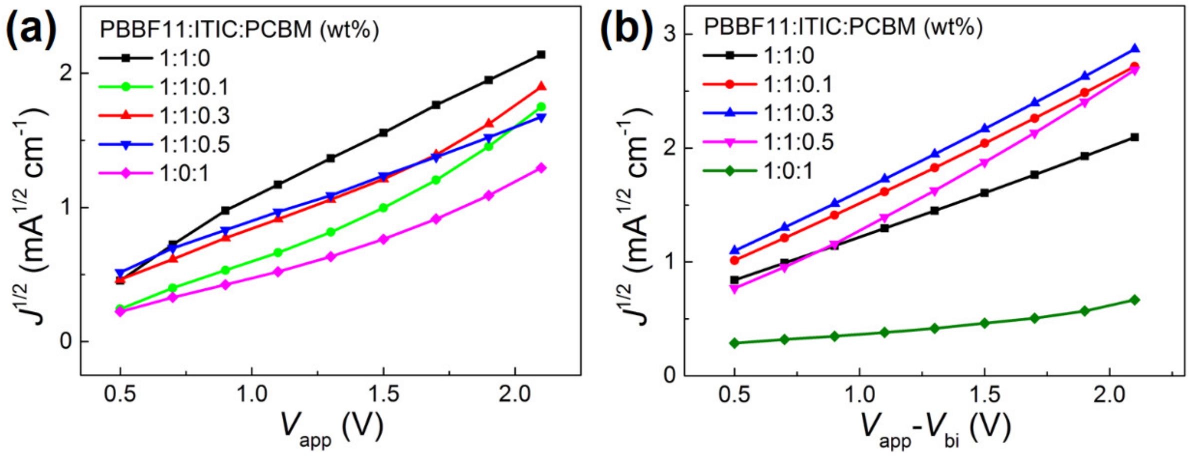

Figure S7. SCLC measurements of the **PBBF11**:ITIC:$PC_{71}BM$ blends in the dark. (**a**) electron-only and (**b**) hole-only devices.

Table S1. The α and J_{ph}/J_{sat} values along with SCLC mobilities of **PBBF11**:ITIC:$PC_{71}BM$ based binary and ternary devices

**PBBF11:ITIC:PC$_{71}$BM (wt%)**	**α**	**J_{ph}/J_{sat}**	**μ_h (cm^2 V^{-1} s^{-1})**	**μ_e (cm^2 V^{-1} s^{-1})**	**μ_h/μ_e**
1:1:0	95.9%	89.1%	3.56×10^{-3}	1.23×10^{-3}	2.89
1:1:0.1	98.0%	90.4%	2.75×10^{-3}	2.28×10^{-3}	1.21
1:1:0.3	99.2%	93.0%	2.47×10^{-3}	2.46×10^{-3}	1.00
1:1:0.5	98.1%	90.1%	1.67×10^{-3}	2.96×10^{-3}	0.56
1:0:1	96.8%	86.3%	1.41×10^{-3}	9.96×10^{-3}	0.14

TRPL studies: To unravel the detailed charge carrier dynamics and elucidate the function of $PC_{71}BM$ addition in **PBBF11**:ITIC based OSCs, time-resolved photoluminescence (TRPL) measurements were further conducted as shown in Figure S8. It is clearly observed the average PL lifetimes of neat films are much shorter than the blend films, i.e., $\tau_{PBBF11} < \tau_{ITIC} < \tau_{PBBF11:ITIC:PCBM}$. This provides a direct evidence of charge separation in the blend films compared with neat samples. However, the effect of $PC_{71}BM$ addition cannot be well resolved with the PL lifetime of **PBBF11**:ITIC binary film close to that of the **PBBF11**:ITIC:$PC_{71}BM$ ternary blends. This is because that in the TRPL measurements, PL decay only reflects the

dynamic related to the emissive states and provides only collective information without spectral resolution.

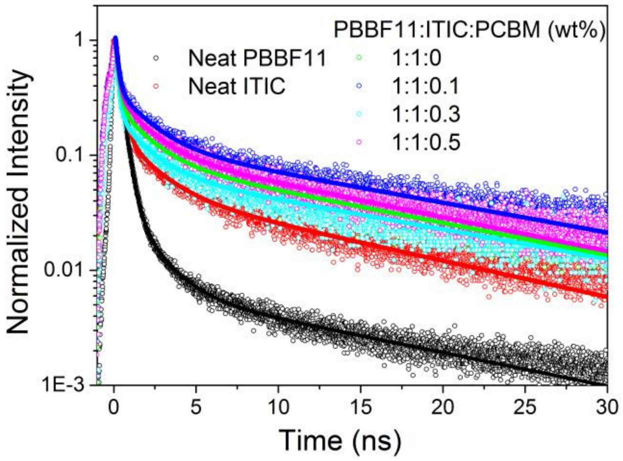

Figure S8. TRPL decays of **PBBF11**, ITIC neat films and blend films excited at 438 nm with 1×10^{12} ph/(cm^2 pulse).

References

[S1] T. Yang, W. Wen, G. Yin, X. Li, M. Gao, Y. Gu, L. Li, Y. Liu, H. Lin, X. Zhang, B. Zhao, T. Liu, Y. Yang, Z. Li, X. Zhou, X. Gao, Nucl. Sci. Tech. **2015**, 26, 020101.

[S2] X. Liu, J. Wang, J. Peng, Z. Liang, Macromolecules **2017**, 50, 6954–6960.

[S3] H. Lu, J. Zhang, J. Chen, Q. Liu, X. Gong, S. Feng, X. Xu, W. Ma, Z. Bo, Adv. Mater. **2016**, 28, 9559–9566.

[S4] J. Sun, X. Ma, Z. Zhang, J. Yu, J. Zhou, X. Yin, L. Yang, R. Geng, R. Zhu, F. Zhang, W. Tang, Adv. Mater. **2018**, 30, 1707150.

[S5] S. Chen, H. Yao, Z. Li, O. M. Awartani, Y. Liu, Z. Wang, G . Yang, J. Zhang, H. Ade, H. Yan, Adv. Energy Mater. **2017**, 7, 1602304.

[S6] B. A. Gregg, J. Phys. Chem. Lett. **2011**, 2, 3013–3015.
